# Lack of a surface layer in *Tannerella forsythia* mutants deficient in the type IX secretion system

**DOI:** 10.1099/mic.0.080192-0

**Published:** 2014-10

**Authors:** Yuka Narita, Keiko Sato, Hideharu Yukitake, Mikio Shoji, Daisuke Nakane, Keiji Nagano, Fuminobu Yoshimura, Mariko Naito, Koji Nakayama

**Affiliations:** 1 Division of Microbiology and Oral Infection, Department of Molecular Microbiology and Immunology, Nagasaki University Graduate School of Biomedical Sciences, Nagasaki 819-8588, Japan; 2 Department of Microbiology, School of Dentistry, Aichi Gakuin University, Nagoya, Japan

## Abstract

*Tannerella forsythia*, a Gram-negative anaerobic bacterium, is an important pathogen in periodontal disease. This bacterium possesses genes encoding all known components of the type IX secretion system (T9SS). *T. forsythia* mutants deficient in genes orthologous to the T9SS-encoding genes *porK*, *porT* and *sov* were constructed. All *porK*, *porT* and *sov* single mutants lacked the surface layer (S-layer) and expressed less-glycosylated versions of the S-layer glycoproteins TfsA and TfsB. In addition, these mutants exhibited decreased haemagglutination and increased biofilm formation. Comparison of the proteins secreted by the *porK* and WT strains revealed that the secretion of several proteins containing C-terminal domain (CTD)-like sequences is dependent on the *porK* gene. These results indicate that the T9SS is functional in *T. forsythia* and contributes to the translocation of CTD proteins to the cell surface or into the extracellular milieu.

## Introduction

Oral biofilms comprise more than 700 bacterial species and matrix substances and contribute to the development of periodontal disease (Aas *et al.*, 2005). Although chronic periodontitis is caused by a mixed infection, specific micro-organisms including *Porphyromonas gingivalis*, *Tannerella forsythia* and *Treponema denticola* are considered important for the initiation and progression of chronic periodontitis (Holt & Ebersole, 2005).

*P. gingivalis* encodes a variety of virulence factors, such as the extracellular and cell-surface cysteine proteinases Arg-gingipain and Lys-gingipain (Potempa *et al.*, 2003; O'Brien-Simpson *et al.*, 2003). Recently, these proteinases were shown to be secreted by the Por secretion system (PorSS) (Sato *et al.*, 2005; Sato *et al.*, 2010). The proteins constituting the PorSS differ from those constituting other secretion systems. The *P. gingivalis* PorSS includes the PorK, PorL, PorM, PorN, PorP, PorQ, PorT, PorU, PorV (PG27, LptO), PorW and Sov proteins (Sato *et al.*, 2010). Coding sequences (CDSs) encoding proteins homologous to the *P. gingivalis* PorSS proteins are present in the genomes of several bacteria in phylum *Bacteroidetes* (McBride & Zhu, 2013). Therefore, the PorSS has been called the type IX secretion system (T9SS) (Chagnot *et al.*, 2013).

*T. forsythia*, an anaerobic Gram-negative bacterium, belongs to phylum *Bacteroidetes*. It appears to possess the T9SS because it has genes encoding all known components of the T9SS (Sato *et al.*, 2010). *T. forsythia* is phylogenetically related to *P. gingivalis*; however, unlike *P. gingivalis*, *T. forsythia* does not form black-pigmented colonies on blood-agar plates. Mixed infection by *T. forsythia* and *P. gingivalis* enhanced abscess formation in a murine model (Takemoto *et al.*, 1997; Yoneda *et al.*, 2001). *T. forsythia* encodes multiple potential virulence factors, including the PrtH proteinase and surface components such as surface layer (S-layer) glycoproteins (TfsA and TfsB) and the leucine-rich-repeat protein BspA (Sharma, 2010). Some virulence-related proteins, including TfsA, TfsB and BspA, appear to have C-terminal domains (CTDs) that may function as a recognition signal for the T9SS (Veith *et al.*, 2009; Shoji *et al.*, 2011).

In this study, *T. forsythia* mutants deficient in *porK*, *porT* and *sov* orthologous genes that may be involved in the translocation of CTD proteins such as TfsA, TfsB and BspA to the cell surface were generated. The *porK*, *porT* and *sov* mutant cells exhibited morphological changes and expressed less-glycosylated versions of the S-layer proteins TfsA and TfsB. In the *porK* mutant, several CTD proteins were not secreted into the extracellular milieu. These results indicate that the T9SS is functional in *T. forsythia* and is important for the virulence of this bacterium.

## Methods

### 

#### Bacterial strains and culture conditions.

All bacterial strains and plasmids used in this study are listed in Table 1. *T. forsythia* cells were grown anaerobically (10 % CO_2_, 10 % H_2_, and 80 % N_2_) in enriched brain heart infusion broth (BHI) medium (Sato *et al.*, 2010) supplemented with 10 µg ml^−1^ *N*-acetylmuramic acid (MurNAc) (Sigma-Aldrich) and 5 % (v/v) heat-inactivated calf serum (CS) and on enriched tryptic soy agar (Sato *et al.*, 2010) supplemented with 10 µg ml^−1^ MurNAc and 5 % (v/v) defibrinated laked sheep blood. For the selection and maintenance of erythromycin (Em)-resistant *T. forsythia* strains, Em was added to the medium at a concentration of 5 µg ml^−1^.

**Table 1.** Bacterial strains and plasmids used in this study

Strain or plasmid	Description	Reference or source
Bacterial strains		
*Tannerella forsythia*		
43037	WT	ATCC
NTF1	*porK* : : *ermF*, Em^r^	This study
NTF2	*ΔporT* : : *ermF*, Em^r^	This study
NTF3	*sov* : : *ermF*, Em^r^	This study
Δ230	*ΔtfsA* : : *cat*, Cp^r^	Sakakibara *et al.* (2007)
Δ270	*ΔtfsB* : : *cat*, Cp^r^	Sakakibara *et al.* (2007)
Δ230-270	*ΔtfsA tfsB* : : *cat*, Cp^r^	Sakakibara *et al.* (2007)
*Escherichia coli*		
XL-1Blue	Host strain for cloning	Stratagene
Plasmids		
pCR4 Blunt TOPO	Ap^r^, Km^r^, PCR cloning vector	Invitrogen
pBluescript II SK(-)	Ap^r^, cloning vector	Stratagene
pKD1030	Ap^r^, contains *porK*-5′ region in pBluescript II SK(–)	This study
pKD1031	Ap^r^, contains *porK*-5′ and -3′ regions in pBluescript II SK(−)	This study
pKD1032	Ap^r^, contains the *ermF* DNA cassette at the *Bam*HI site of pKD1031	This study
pKD1033	Ap^r^, contains *porT*-upstream region in pCR4 Blunt TOPO	This study
pKD1034	Ap^r^, contains *porT*-upstream and -downstream regions in pCR4 Blunt TOPO	This study
pKD1035	Ap^r^, contains the *ermF* DNA cassette at the *Bam*HI site of pKD1034	This study
pKD1036	Ap^r^, contains 2.0 kb *sov* fragment in pCR4 Blunt TOPO	This study
pKD1037	Ap^r^, contains the *ermF* DNA cassette at the *Bam*HI site of pKD1036	This study

Em^r^, erythromycin resistance; Ap^r^, ampicillin resistance; Cp^r^, chloramphenicol resistance; Km^r^, kanamycin resistance.

#### Construction of bacterial strains.

Genomic nucleotide sequence data of *T. forsythia* ATCC 43037 was obtained from the GenBank database (accession number: CP003191). The *T. forsythia porK* insertion mutant was constructed as follows. A 0.6 kb 5′-terminal region of *porK* was amplified from the chromosomal DNA of *T. forsythia* ATCC 43037 using the Pyrobest DNA polymerase (TaKaRa) and PCR using the primers TFporKUF and TFporKUR. The DNA primers used in this study are listed in Table S1 (available in the online Supplementary Material). The amplified DNA was cloned into the pCR4 Blunt TOPO vector (Invitrogen) according to the manufacturer’s instructions and digested with *Eco*RI and *Bam*HI. The resulting DNA fragment was then inserted into the *Eco*RI and *Bam*HI sites of pBluesript II SK(−) to generate pKD1030. A 0.8 kb 3′-terminal region of *porK* was amplified from the chromosomal DNA of ATCC 43037 with the primers TFporKDF and TFporKDR. The amplified DNA was cloned into pCR4 Blunt TOPO and digested with *Bam*HI and *Not*I. The resulting fragment was then inserted into the *Bam*HI and *Not*I sites of pKD1030 to generate pKD1031. The 1.1 kb *Bam*HI *ermF* DNA cassette was inserted into the *Bam*HI site of pKD1031, resulting in pKD1032 (*porK* : : *ermF*). The pKD1032 was linearized with *Not*I and introduced into ATCC 43037 by electroporation to generate the NTF1 strain.

The *T. forsythia porT* deletion mutant (NTF2) was constructed as described above except that the DNA regions upstream and downstream of *porT* were amplified by PCR from the chromosomal DNA of the strain ATCC 43037 with the primers TFporTUF and TFporTUR and the primers TFporTDF and TFporTDR, respectively.

The *T. forsythia sov* insertion mutant was constructed as follows. A 2.0 kb internal region of the *sov* gene was amplified from the chromosomal DNA of the strain ATCC 43037 by PCR with the primers TFsovF and TFsovR. The amplified DNA was cloned into pCR4 Blunt TOPO to generate pKD1036. The 1.1 kb *Bam*HI *ermF* DNA cassette was inserted into the *Bam*HI site in the *sov* region of pKD1036, resulting in pKD1037 (*sov* : : *ermF*). The pKD1037 was linearized with *Not*I and introduced into *T. forsythia* ATCC 43037 by electroporation to generate the NTF3 strain.

#### Electron microscopy.

To examine bacterial cell shape, the cells were washed and negatively stained on carbon-coated grids with 1 % ammonium molybdate. To prepare ultrathin sections, the cells were fixed with 4 % paraformaldehyde and 5 % glutaraldehyde in 30 mM HEPES buffer (pH 7.4) overnight at 4 °C. The samples were post-fixed with 1 % osmium tetroxide for 2 h and then with 0.5 % uranyl acetate for 30 min. The fixed cells were dehydrated in a series of 25–100 % ethanol and embedded in Quetol-651 resin (Nisshin EM). The ultrathin sections were stained with 1 % uranyl acetate and 1 % lead citrate. The stained samples (bacterial cells and ultrathin sections) were observed using a JEM-1210 transmission electron microscope (JEOL).

#### Gel electrophoresis and immunoblot analysis.

SDS-PAGE and immunoblot analyses were performed as previously described (Shoji *et al.*, 2011). The blotted membranes were treated with anti-TfsA and anti-TfsB antisera (Sakakibara *et al.*, 2007).

The glycoproteins in SDS-PAGE gels were stained using the Pro-Q Emerald 300 fluorescent stain (Invitrogen). After staining with Pro-Q Emerald 300, total protein staining was performed with SYPRO Ruby (Invitrogen).

#### Two-dimensional gel electrophoresis (2D-PAGE).

2D-PAGE was performed as described previously (Sato *et al.*, 2013). *T. forsythia* strains were grown in serum-free medium. Particle-free culture supernatants were obtained as previously described (Sato *et al.*, 2013). The proteins in the particle-free culture supernatant fraction were precipitated with 10 % (w/v) trichloroacetic acid at 4 °C. The precipitated proteins were harvested by centrifugation at 4 °C for 20 min, washed three times with cold diethyl ether, dried at room temperature for 30 min and then resuspended in a cell lysis solution (7 M urea, 2 M thiourea, 4 % CHAPS, 1 mM EDTA and 5 mM tributylphosphine). The samples were applied to 13 cm immobilized pH gradient strips (GE Healthcare Bio-Sciences) with a pH range from 4 to 7 (first dimension) swollen with a rehydration solution [7 M urea, 2 M thiourea, 4 %, v/v, CHAPS, 0.5 %, v/v, IPG buffer pH 4 to 7 (GE Healthcare), 1 mM EDTA, 12 µl ml^−1^ destreak reagent (GE Healthcare)] and bromophenol blue. The 2D electrophoresis (SDS-PAGE) was performed in polyacrylamide gels, and the proteins were stained with Coomassie brilliant blue (CBB) R250 .

#### MS analysis and database search for protein identification.

Proteins were identified by peptide-mass fingerprinting after in-gel tryptic digestion as previously described (Sato *et al.*, 2010). Gel plugs containing proteins were subjected to washing with 50 % (v/v) acetonitrile, washing with 100 % acetonitrile, reduction with 10 mM DTT (Wako), alkylation with 55 mM iodoacetamide, washing/dehydration with 50 % (v/v) acetonitrile and digestion for 10 h with 10 µg ml^−1^ trypsin. The resulting peptides were extracted from the gel plugs with 0.1 % (v/v) trifluoroacetic acid and 50 % (v/v) acetonitrile. The digests were spotted on a MALDI target using α-cyano-4-hydroxycinnamic acid as a matrix. The spectra were acquired on a 4800 MALDI TOF/TOF Analyser (Applied Biosystems). Data analysis and MS database searching were performed using GPS Explorer and Mascot software (Matrix Science) with the significance criteria of the program (*P*<0.05).

#### Haemagglutination analysis.

Haemagglutination was assessed as previously described (Shoji *et al.*, 2013).

#### Assay for trypsin-like activity.

Trypsin-like activity was assayed as described previously (Grenier, 1995). Trypsin-like activity was measured by monitoring the hydrolysis of the chromogenic synthetic peptide benzoyl-dl-arginine-*p*-nitroanilide (BAPNA; Peptide Institute) in the presence or absence of various compounds: EDTA (Wako Pure Chemical Industries), N-α-p-tosylamide-2-phenylethyl chloromethyl ketone (TLCK; Wako), leupeptin (Peptide Institute), DTT, iodoacetamide (Wako), SDS (Wako), CaCl_2_ (Sigma-Aldrich), MgCl_2_ (Wako) and ZnCl_2_ (Wako). The cells were harvested by centrifugation (10 000 ***g***, 30 min) and suspended in distilled water. The bacterial samples (12.5 µl) were mixed with 125 µl of 150 mM Tris/HCl buffer (pH 7.8), 50 µl of 4 mM BAPNA and 12.5 µl distilled water, and the assay mixtures were incubated at 37 °C for 2 h. The release of *p*-nitroaniline was determined by measuring the OD_405_ nm using a microplate reader (Bio-Rad).

#### Biofilm formation.

Biofilm formation was measured by a microtitre plate biofilm assay using a previously reported protocol with slight modification (O'Toole & Kolter, 1998; Honma *et al*., 2007). Briefly, an overnight culture adjusted to an OD_595_ nm of 1.0 was diluted 1 : 10 with fresh medium. The cells were aliquoted into the wells of a 96-well microtitre plate (250 µl per well) and incubated anaerobically for 1–4 days. After removal of the planktonic cells by washing twice with PBS, the biofilm was stained by incubation with 100 µl of 0.1 % (w/v) crystal violet solution for 5 min. The plate was washed twice with distilled water and destained with 200 µl of 95 % (v/v) ethanol for 5 min. Biofilm mass was evaluated at OD_595_ nm at 1, 2, 3 and 4 days using a microplate reader. Total biofilm formation was evaluated as the absorbance of crystal violet stained biofilms at OD_595 nm_ divided by the absorbance of total growth (including biofilm and planktonic cells) at OD_595_ nm.

For visualization by microscopy, *T. forsythia* biofilms were formed in 4-well Lab Tek II chamber slides (Nunc) as described previously (Honma *et al.*, 2007). The diluted cells described above were dispensed in each chamber and incubated anaerobically for 3 days. Following incubation, chambers were washed with PBS and biofilms were stained with the SYTO9 LIVE/DEAD Baclight Bacterial Viability kit L7012 (Invitrogen, Molecular Probe). The stain was prepared according the manufacturer’s instruction and coverslips mounted using Vector Shield (Vector Laboratories). The edges of the coverslips were coated with nail polish and the slide was kept in the dark until analysed. Microscope images of fluorescence were examined using ZEISS Axioskop plus microscope mounted with an AxioCam HRc camera with a Plan-Neofluar 20× 0.5 NA objective (ZEISS). The Z-stack images of the biofilm were acquired with confocal laser scanning microscope (LSM510 META, AxioVert200M) with a LD-Achroplan 40× 0.6 NA objective (ZEISS). For three-dimensional reconstruction, the image analysis was done using Z-series image stacks of each biofilm with the LSM image browser and ZEN2009 software.

#### Statistical analysis.

The data are presented as means±sd. The biofilm formation data were analysed using one-way ANOVA with the Tukey–Kramer multiple-comparison test. *P*-values less than 0.05 were considered significant.

## Results

### Construction of *T. forsythia* mutants deficient in T9SS proteins

The erythromycin-resistance DNA cassette was inserted into the *T. forsythia* CDSs bfor_c_1_3635, bfor_c_1_6468 and bfor_c_1_12435 (locus tags by the Human Oral Microbiome Database), which were orthologous to *P. gingivalis porK*, *porT* and *sov*, to generate *T. forsythia porK* (NTF1), *porT* (NTF2) and *sov* (NTF3) mutants, respectively (Fig. S1).

### Cell morphology of the *porK*, *porT* and *sov* mutants

**Fig. 1.**
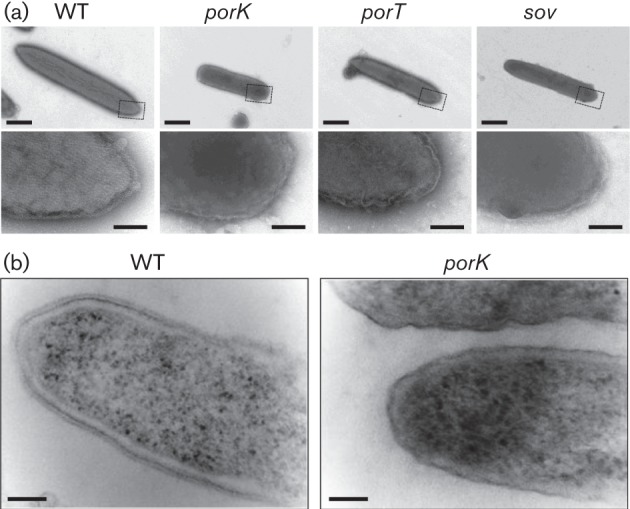
Comparison of the cell shape of WT *T. forsythia* and T9SS-deficient mutants. (a) Electron micrographs of negatively stained cells. The regions indicated by dashed boxes in the upper panels are magnified in the lower panels. Bars, 500 nm (upper), 100 nm (lower). (b) Electron micrographs of ultrathin cell sections. Bars, 100 nm.

The bacterial cells were negatively stained with ammonium molybdate and analysed by electron microscopy. WT *T. forsythia* cells were prolate, ellipsoid-like with sharp ends, whereas the *porK*, *porT* and *sov* mutants were rod-like with round ends (Fig. 1a). The WT cells showed a lattice structure on the cell surface, whereas the *porK*, *porT* and *sov* mutants exhibited amorphous and fragile surfaces. The lattice structure of the WT *T. forsythia* cell surface is the S-layer, which contains the TfsA and TfsB proteins (Sakakibara *et al.*, 2007; Sekot *et al.*, 2012); therefore, the *porK*, *porT* and *sov* mutants appeared to lack the S-layer. Analysis of the ultrathin cross-sections revealed that the WT cells had an S-layer with a thickness of approximately 20 nm on the outer membrane, whereas the *porK* mutant cells did not possess this structure on the outer membrane (Fig. 1b).

### SDS-PAGE and immunoblot analyses of whole-cell lysates

**Fig. 2.**
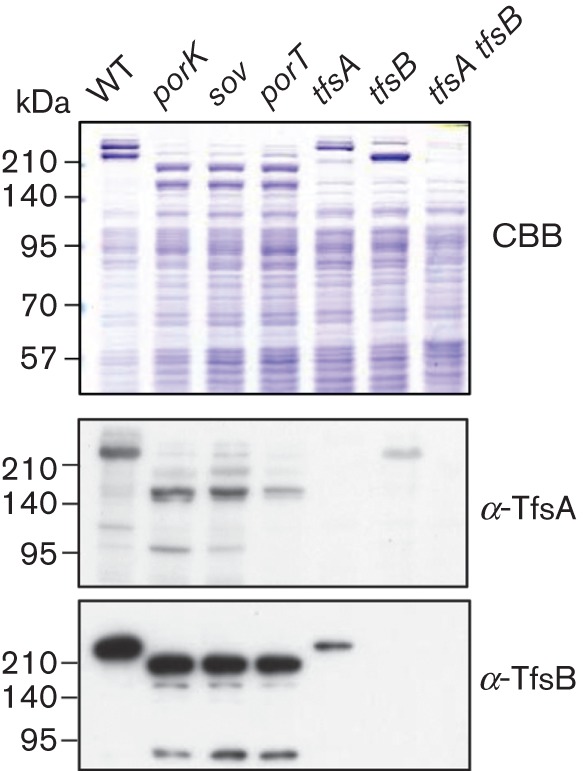
SDS-PAGE and immunoblotting analysis. *T. forsythia* cell lysates were analysed by SDS-PAGE and immunoblotting using anti-TfsA and anti-TfsB antisera. The proteins were stained with CBB R250.

**Fig. 3.**
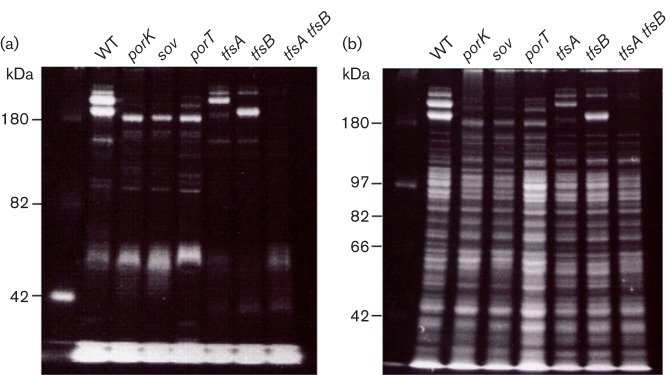
Glycoprotein staining of SDS-PAGE gels of *T. forsythia* cells with (a) Pro-Q Emerald 300 fluorescent glycoprotein stain and (b) SYPRO Ruby fluorescent total protein stain. The first lane in each gel contains the CandyCane glycoprotein molecular mass standards (Invitrogen), a mixture of glycosylated and non-glycosylated proteins used as a positive control for staining.

The SDS-PAGE profiles of whole-cell lysates revealed that major proteins with molecular masses of 230 and 270 kDa in the WT cells were not present in the *porK*, *porT* or *sov* mutant cells; furthermore, the *porK*, *porT* and *sov* mutant cells contained major proteins with molecular masses of 165 and 205 kDa that were absent in the WT cells (Fig. 2). Immunoblotting analysis using antisera against the TfsA and TfsB proteins revealed that the 230 and 270 kDa proteins in the WT cells were the TfsA and TfsB proteins, respectively, and that the 165 and 205 kDa proteins in the *porK*, *porT* and *sov* mutants were derived from the TfsA and TfsB proteins, respectively. The molecular masses of the TfsA and TfsB proteins without signal peptides are 133.3 kDa and 150.8 kDa, respectively (Lee *et al.*, 2006); therefore, it was examined whether these proteins were glycosylated (Fig. 3). ProQ-Emerald carbohydrate staining analysis suggested that the 230 kDa TfsA protein and the 270 kDa TfsB protein were strongly glycosylated in WT cells and that the 165 kDa TfsA protein and 205 kDa TfsB protein were also glycosylated in the *porK*, *porT* and *sov* mutants, albeit to a lesser extent.

### 2D-PAGE analysis of particle-free culture supernatants

**Fig. 4.**
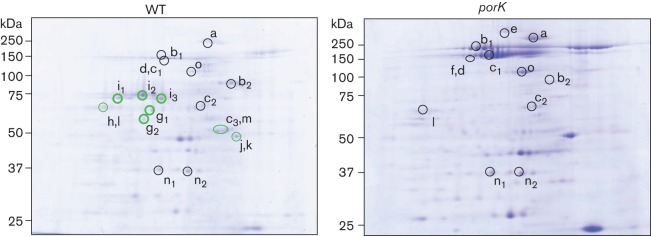
2D gel analysis. 2D-PAGE analysis was performed using IPG strips with a pH 4–7 isoelectric point range to analyse the particle-free culture supernatants of the WT and NTF1 (*porK*) strains. The proteins were stained with CBB R250. Green circles indicate the spots with no, or lower, protein levels in the *porK* mutant cells compared with the WT cells. Identification of protein spots in Fig. 4 is provided in Table 2.

2D-PAGE was performed to analyse the particle-free (membrane-free) culture supernatants from the WT and *porK* strains (Fig. 4). Time of flight mass spectrometry (TOFMS) analysis revealed the presence of the *T. forsythia* proteins bfor_c_1_1931 (tentatively named type Nine Secretion System-dependent protein A, NdpA), bfor_c_1_8519 (NdpB), bfor_c_1_10593 (NdpC), bfor_c_1_10600 (NdpD) and bfor_c_1_14540 (NdpE) in the particle-free culture supernatant of the WT cells but not the *porK* mutant cells (Table 2).

**Table 2.** Identification of protein spots in 2D-gels

Protein spot		HOMD name	Description	Mascot score	
WT	NTF1
a	*	bfor_c_1_3670	Hypothetical protein	261	616
b_1_	*	bfor_c_1_4507	Hypothetical protein	71	1222
b_2_	*	bfor_c_1_4507	Hypothetical protein	456	162
c_1_	*	bfor_c_1_4502	Hypothetical protein	559	419
c_2_	*	bfor_c_1_4502	Hypothetical protein	257	181
c_3_	*	bfor_c_1_4502	Hypothetical protein	26	–
c_1_	*	bfor_c_1_4505	Hypothetical protein	277	658
d	*	bfor_c_1_4347	Conserved hypothetical protein	389	266
e	*	bfor_c_1_629	Hypothetical protein	–	165
f	*	bfor_c_1_14680	Conserved hypothetical protein; possible haemagglutinin/haemolysin	–	900
g_1_	*	bfor_c_1_8519	PorU, conserved hypothetical protein	272	-
g_2_	*	bfor_c_1_8519	PorU, conserved hypothetical protein	200	-
h	†	bfor_c_1_14540	Thermolysin precursor	216	-
i_1_	†	bfor_c_1_10600	Lysyl endopeptidase	425	-
i_2_	†	bfor_c_1_10600	Lysyl endopeptidase	96	-
i_3_	†	bfor_c_1_10600	Lysyl endopeptidase	300	-
j	†	bfor_c_1_10593	Eukaryotic-like metalloproteinase, karilysin	84	-
k	†	bfor_c_1_1931	Thermolysin; zinc metalloprotease	44	-
l	‡	bfor_c_1_843	Possible lipoprotein	107	264
m	‡	bfor_c_1_2071	Conserved hypothetical protein	182	-
n_1_	‡	bfor_c_1_2868	Conserved hypothetical protein	357	410
n_2_	‡	bfor_c_1_2868	Conserved hypothetical protein	449	341
o		bfor_c_1_4765	Zinc protease	129	594

HOMD, Human Oral Microbiome Database.

* CTD family protein (Veith *et al.*, 2009).

† Probable CTD family protein.

‡ Antigenic protein (non-CTD) (Veith *et al.*, 2009).

### Haemagglutination

**Fig. 5.**
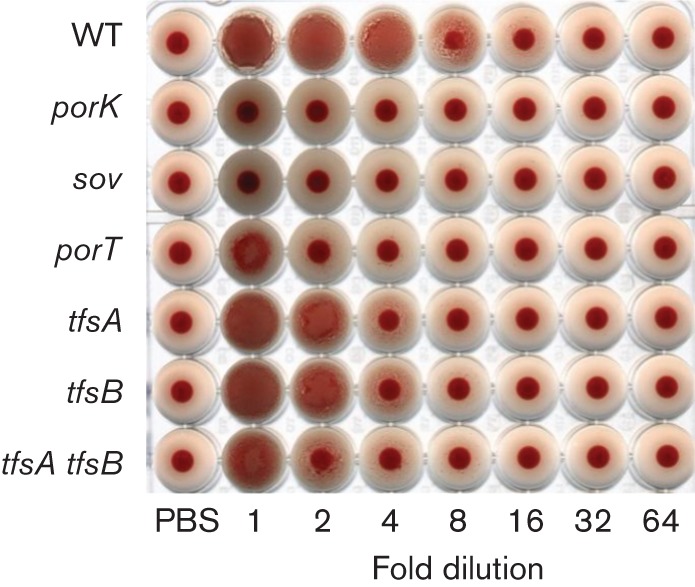
Haemaggulutination. Suspensions of *T. forsythia* cells and serial twofold dilutions in PBS were applied to the wells of a microtitre plate from left to right and mixed with a sheep erythrocyte suspension.

Purified S-layers cause erythrocyte agglutination, and S-layer-deficient mutant strains exhibit decreased haemagglutination activity (Sabet *et al.*, 2003; Sakakibara *et al.*, 2007). The haemagglutination activities of the *porK*, *porT* and *sov* mutants on sheep erythrocytes were measured (Fig. 5). The haemagglutination activities of the *porK*, *porT* and *sov* mutants were 12.5 % lower compared with the WT strain. The haemagglutination activities of the *tfsA* and *tfsB* single mutants were 50 % of the WT level, whereas the haemagglutination activity of the *tfsA tfsB* double mutant cells was 25 % of the WT level.

### Biofilm formation

**Fig. 6.**
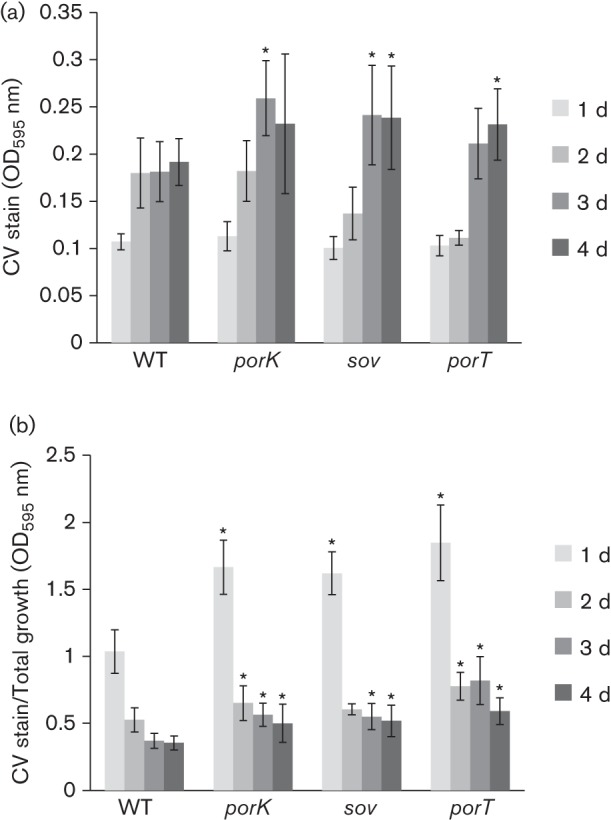
Biofilm-forming ability. (a) The adherent cells formed after 1, 2, 3 and 4 days were quantified by measuring the OD_595_ nm of crystal violet (CV). (b) OD_595_ nm of CV staining/OD_595_ nm of total bacterial growth. Three independent experiments were performed in quadruplicate or quintuplicate to provide 13 sets of data. The differences in each strain compared with the WT were analysed using one-way ANOVA with the Tukey–Kramer multiple-comparison test. *P<0.01. WT, *T. forsythia* ATCC 43037; *porK*, NTF1; *porT*, NTF2; *sov*, NTF3.

**Fig. 7.**
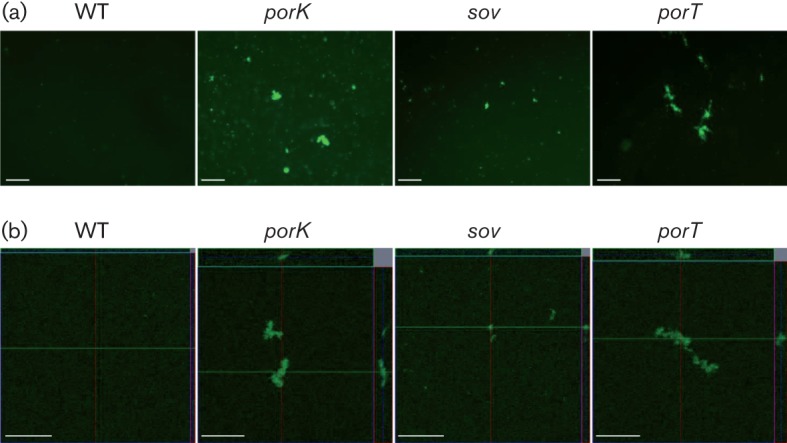
Biofilm analysis by fluorescence microscopy (FM) and confocal laser scanning microscopy (CLSM). Bacterial cells were stained with SYTO 9. (a) FM images of 3 day biofilms. Bars, 50 µm. (b) CLSM images of 3 day biofilms. Biofilm thicknesses of each strain are: WT (3.86 µm), *porK* (22.4 µm), *sov* (8.5 µm), *porT* (14.7 µm). x-z and y-z reconstructions of each biofilm are shown upper and right sides of x-y image. Bars, 50 µm.

The *T. forsythia wecC* mutant strain, which lacks UDP-*N*-acetylmannosaminuronic acid dehydrogenase and has truncated S-layer glycans, shows increased biofilm formation (Honma *et al.*, 2007; Posch *et al.*, 2011). An examination of the biofilm-forming ability of the *porK*, *porT* and *sov* mutants (Figs 6 and 7) revealed that these mutants showed increased biofilm formation.

### Trypsin-like activity

*T. forsythia* produces an enzymic endopeptidase that degrades BAPNA; this endopeptidase activity was attributed to a trypsin-like proteinase (Grenier, 1995). To examine whether the *porK*, *porT* and *sov* mutations affect the trypsin-like endopeptidase activity of *T. forsythia*, BAPNA hydrolysis using *porK*, *porT* and *sov* mutant cells in the presence and absence of various chemicals including protease inhibitors was monitored (Table 3). The hydrolysis activities of the *porK*, *porT* and *sov* mutant cells were similar to that of the WT. The BAPNA-hydrolysis activities of the mutant and WT cells were completely or partially suppressed by TLCK, ZnCl_2_ and leupeptin, indicating that the WT and mutant cells contained similar BAPNA-hydrolysis properties.

**Table 3.** Trypsin-like activity

Inhibitor	Concn. (final)	Residual activity (%)*			
WT	*porK*	*porT*	*sov*
None	–	100	127.7	126.9	110.4
EDTA	10 mM	107.6	123.1	126.9	105.7
Iodoacetamide	10 mM	113.7	122.3	155.7	101.3
TLCK	1 mM	1.3	4.0	7.3	0.7
Leupeptin	0.01 mM	57.5	72.3	78.1	52.2
CaCl_2_	10 mM	87.3	103.3	122.5	91.7
ZnCl_2_	10 mM	28.6	14.2	28.9	19.6
DTT	10 mM	112.3	128.8	99.8	113.7

* Trypsin-like activities of various *T. forsythia* strains with various protease inhibitors and metal ions were determined using BAPNA as a substrate. Enzymic activity of the WT without any additive in the reaction mixture was taken as 100 %.

## Discussion

The T9SS (PorSS) was discovered in the periodontal pathogen *P. gingivalis* (Sato *et al.*, 2010). Subsequently, homologous genes encoding putative T9SS components were observed in several bacterial species of phylum *Bacteroidetes*, suggesting that the T9SS is conserved in at least a subset of this phylum (McBride & Zhu, 2013; Chagnot *et al.*, 2013). The T9SS is related to gliding motility of bacteria of phylum *Bacteroidetes* (Sato *et al.*, 2010; Nakane *et al.*, 2013).

*T. forsythia*, which belongs to phylum *Bacteroidetes*, is a member of the ‘red complex’ together with *P. gingivalis* and *T. denticola* and is considered a major pathogen underlying periodontal disease. *T. forsythia* possesses several putative virulence factors such as trypsin-like protease, PrtH protease, sialidases, BspA leucine-rich repeat protein and the S-layer. In this study, we generated three T9SS-deficient *T. forsythia* mutants in which the *porK*, *porT* and *sov* genes were mutated. All mutations caused identical phenotypes such as absence of the S-layer, decreased haemagglutination activity and increased biofilm formation, suggesting that these properties are related to the T9SS.

The S-layer is a paracrystalline surface-protein array expressed in several bacteria and is thought to function as a protective coat against external sieves and ion traps (Sleytr & Beveridge, 1999; Sabet *et al.*, 2003; Messner *et al.*, 2010). The *T. forsythia* S-layer mediates adhesion to human gingival epithelial cells and subsequent invasion (Sakakibara *et al.*, 2007) and delays recognition of the bacterium by the host innate immune system (Sekot *et al.*, 2011). The S-layer contributes to *T. forsythia* serum resistance and oral bacterial coaggregation (Shimotahira *et al.*, 2013) and consists of the TfsA and TfsB proteins. S-layer proteins generally contain N-terminal signal peptides, with a few exceptions (Boot & Pouwels, 1996). Because the primary products of the *tfsA* and *tfsB* CDSs contain N-terminal signal peptides, these proteins are probably translocated across the inner membrane by a Sec-dependent mechanism. The TfsA and TfsB proteins contain CTD-like sequences at the C terminus, and CTD sequences are a signal for T9SS-mediated translocation across the outer membrane (Shoji *et al.*, 2011), suggesting that the S-layer proteins are translocated across the outer membrane by the T9SS. Posch *et al.* (2013) observed that the molecular masses of His-tagged TfsA and TfsB proteins expressed in *Bacteroides fragilis,* which are probably located in the periplasm because of lack of T9SS in *B. fragilis* (Sato *et al.*, 2010; McBride & Zhu, 2013), are ~170 kDa and ~200 kDa, respectively, which are consistent with the molecular masses of the TfsA and TfsB proteins in the *T. forsythia* T9SS-deficient mutants. *T. forsythia* as well as *B. fragilis* has an *O*-glycosylation system (Fletcher *et al.*, 2009; Coyne *et al.*, 2013; Posch *et al.*, 2011, 2013). These findings suggest that in *T. forsythia* cells, the S-layer proteins are primarily *O*-glycosylated at the inner membrane and/or in the periplasm, and after translocation across the outer membrane by the T9SS, the proteins are further glycosylated on the cell surface.

Using 2D-gel analysis, we observed that the NdpA, NdpB, NdpC, NdpD and NdpE proteins were released into the WT but not the *porK* culture supernatant. The NdpA and NdpE proteins are thermolysin metallopeptidase homologues; the NdpB protein is a PorU homologue (Sato *et al.*, 2010; Glew *et al.*, 2012); the NdpC protein is a karilysin (Karim *et al.*, 2010) and the NdpD protein is a putative lysyl endopeptidase homologous to *P. gingivalis* PepK (Nonaka *et al.*, 2014). These proteins are putative peptidases and possess CTD-like sequences at their C termini. These results indicate that the T9SS is functional in *T. forsythia* and is involved in the secretion of CTD proteins.

Sabet *et al.* (2003) purified the S-layer from *T. forsythia* and observed that the S-layer was sufficient to mediate the haemagglutination of sheep erythrocytes. Sakakibara *et al.* (2007) generated *tfsA* and *tfsB* single mutants and a *tfsA tfsB* double mutant and observed that these S-layer-deficient mutants caused decreased haemagglutination of chicken erythrocytes. In this study, it was observed that the *porK*, *porT* and *sov* mutants caused decreased haemagglutination of sheep erythrocytes. The haemagglutination activity of the T9SS-deficient mutants was weaker than that of the S-layer-deficient mutants, suggesting that cell-surface proteins other than TfsA and TfsB, which are secreted by the T9SS, are also involved in haemagglutination.

Honma *et al.* (2007) isolated a *wecC* mutant that showed increased biofilm formation and observed that the molecular masses of both S-layer proteins were decreased in the *wecC* mutant. Subsequently, the decreased molecular mass of the S-layer proteins was correlated with truncated S-layer glycans (Posch *et al.*, 2011). In this study, it was observed that the T9SS-deficient mutants lacked S-layers and contained TfsA and TfsB proteins with decreased molecular masses and reduced glycosylation compared with WT cells; these mutants also showed increased biofilm formation. These results indicate that S-layers or S-layer glycans suppress *T. forsythia* biofilm formation.

Trypsin-like endopeptidase activity was observed in the *T. forsythia* cell envelope (Grenier, 1995). The T9SS-deficient mutants showed the same trypsin-like activity as the WT, suggesting that the T9SS is not required for translocation of the trypsin-like enzyme(s) to the cell envelope.

In this study, *T. forsythia porK, porT* and *sov* mutant strains were generated, and these mutants were found to lack the S-layer. Several CTD proteins such as thermolysin were not observed in the culture supernatant of the *porK* mutant cells. These results indicate that the T9SS is functional in *T. forsythia* and contributes to translocation of the CTD proteins to the cell surface or into the extracellular milieu.
